# Astaxanthin: A Marine Drug That Ameliorates Cerebrovascular-Damage-Associated Alzheimer’s Disease in a Zebrafish Model via the Inhibition of Matrix Metalloprotease-13

**DOI:** 10.3390/md21080433

**Published:** 2023-07-31

**Authors:** Nallupillai Paramakrishnan, Khian Giap Lim, Yamunna Paramaswaran, Nemat Ali, Mohammad Waseem, Gamal A. Shazly, Yousef A. Bin Jardan, Arunachalam Muthuraman

**Affiliations:** 1 Department of Pharmacognosy, JSS College of Pharmacy, Mysore 643001, Karnataka, India; 2 Pharmacology Unit, Faculty of Pharmacy, AIMST University, Semeling, Bedong 08100, Kedah, Malaysia; 3 Department of Pharmacology and Toxicology, College of Pharmacy, King Saud University, P.O. Box 2457, Riyadh 11451, Saudi Arabia; 4 School of Pharmacy, University of Maryland Eastern Shore, Princess Anne, MD 21853, USA; 5 Department of Pharmaceutics, College of Pharmacy, King Saud University, Riyadh 11451, Saudi Arabia

**Keywords:** acetylcholinesterase, blood–brain barrier, cerebrovascular disease, streptozotocin, vascular dementia, Lewy bodies

## Abstract

Alzheimer’s disease (AD) is a major type of dementia disorder. Common cognitive changes occur as a result of cerebrovascular damage (CVD) via the disruption of matrix metalloproteinase-13 (MMP-13). In diabetic cases, the progress of vascular dementia is faster and the AD rate is higher. Patients with type 2 diabetes are known to have a higher risk of the factor for AD progression. Hence, this study is designed to investigate the role of astaxanthin (AST) in CVD-associated AD in zebrafish via the inhibition of MMP-13 activity. CVD was developed through the intraperitoneal and intracerebral injection of streptozotocin (STZ). The AST (10 and 20 mg/L), donepezil (1 mg/L), and MMP-13 inhibitor (i.e., CL-82198; 10 μM) were exposed for 21 consecutive days in CVD animals. The cognitive changes in zebrafish were evaluated through light and dark chamber tests, a color recognition test, and a T-maze test. The biomarkers of AD pathology were assessed via the estimation of the cerebral extravasation of Evans blue, tissue nitrite, amyloid beta-peptide aggregation, MMP-13 activity, and acetylcholinesterase activity. The results revealed that exposure to AST leads to ameliorative behavioral and biochemical changes. Hence, AST can be used for the management of AD due to its multi-targeted actions, including MMP-13 inhibition.

## 1. Introduction

Alzheimer’s disease (AD) is a major type of dementia disorder. Vascular dementia (VaD) progression is the next level of dementia disorders. In both cases, common changes are cerebrovascular changes via the disruption of matrix metalloproteinase-13 (MMP-13)-mediated blood–brain barrier (BBB) damage [1]. Subsequently, the reduced cerebral blood flow causes brain tissue damage. Further, it affects the thinking process and the ability to perform everyday tasks. Vascular brain changes coexist with changes in dementia and AD via Lewy body formation. In diabetic cases, the progress of VaD is faster and the AD rate is higher. Patients with type 2 diabetes are known to have a higher risk of the factor for AD progression, whereas patients with type 1 diabetes are more prone to developing the VaD type of dementia. MMPs such as MMP-3, MMP-9, MMP-12, and MMP-13 greatly contribute to the progression of CVD and neurodegeneration [2]. Clinically, MMP-9 and MMP-13 contribute to brain damage via alterations in BBB functions in human stroke patients [3,4].

**Figure 1:**
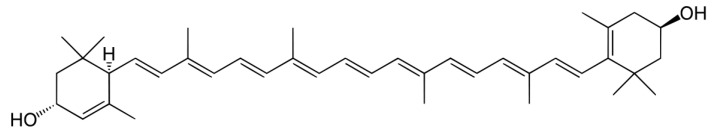
Chemical structure of AST.

Alzheimer’s disease is one of the major neurodegenerative disorders that leads to altered cognitive function due to the aggregation of beta-amyloid peptide and tau protein accumulation in neuronal tissue [5]. Experimentally, medicinal plants are known to regulate the beta-amyloid and tau protein aggregation associated with CVD and AD [5]. CVD is known to cause neurodegeneration and mimic AD pathology. Various natural compounds, such as sinapic acid, auraptene, 4,60-anhydrooxysporidinone [6], rosmarinic acid [7], taurine [8], and epigallocatechin [9], are known to attenuate CVD and AD by improving BBB function and maintaining BBB integrity [9,10]. AST is a xanthophyll carotenoid, and is found in various microorganisms (*Paracoccus carotinifaciens*), microalgae (*Haematococcus* species; genus of *Chlorophycecae*), yeast (*Xanthophyllomyces dendrorhous*), and marine animals, such as salmon (genus *Oncorhynchus*; *Salmo salar*), shrimp (*Penaeus semisulcatus*), krill (*Euphausia pacifica*), and crayfish [11,12,13,14,15]. AST is a lipid-soluble carotenoid, and has variable oral bioavailability due to the absorption of AST by epithelial cells in the small intestine. This absorption value varies based on meal (before or after) intake [16]. Furthermore, the absorption is higher with dietary fat due to the rapid hydrolysis by cholesterol esterase. The maximum plasma concentration and peak plasma concentration were 1.3 ± 0.1 mg/L and 6.7 ± 1.2 h, respectively. Similarly, the volume of distribution and oral clearance were 2.0 ± 1.3 L/kg and 3.3 ± 1.1 L/hour, respectively [17]. AST is safe at a dose of 4–18 mg daily for 12 weeks. However, at a higher dose, it caused stomach pain and red stool [18]. It attenuated neuronal damage against the excitotoxins, i.e., rotenone [19] and 1-methyl-4-phenyl-1,2,3,6-tetrahydropyridine (MPTP) [20]. AST also attenuates the cerebrovascular tissue occlusion, i.e., unilateral common carotid arteries’ occlusion induced VaD via the regulation of interleukin-1β (i.e., IL-1β) and interleukin-4 (IL-4) protein upregulation, superoxide dismutase activity, and lipid peroxidation of the hippocampal and pre-frontal cortex tissue [21]. The chemical structure of AST is illustrated in Figure 1.

Furthermore, AST is attenuated in middle cerebral-artery-occlusion-associated cerebral infarction via the inhibition of cyclic-adenosine-monophosphate-associated protein kinase A activation in mice brains [22]. Hence, AST plays a putative role in the management of neuroinflammation and has significant therapeutic potential for CVD and neurodegenerative disorders [23]. In addition, the molecular modeling study of AST is predicted to undergo non-covalent binding to matrix metalloproteinase-13 (MMP-13) [24]. Moreover, AST declines the upregulation of MMP-1, MMP-3, and MMP-13 [25]. AST also ameliorates diabetic complications, i.e., the neurovascular inflammation of retinal tissue in rats [26], and has a great impact on the pathogenesis of diabetes and its complications [27]. However, the role of AST in diabetes-associated CVD and the mitigating effect of AD via the inhibition of MMP-13 activity have not been studied to date. Hence, this study is focused on evaluating the role of astaxanthin (AST) on the cerebrovascular damage (CVD) associated with AD in zebrafish via the inhibition of MMP-13 activity. 

## 2. Results

### 2.1. Effect of AST on STZ-Induced Diabetic CVD-Associated AD in Light and Dark Chamber Test Response Changes

**Figure 2:**
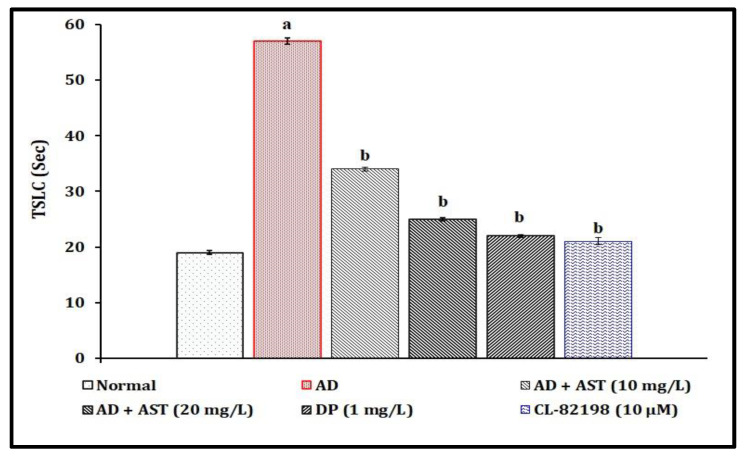
Role of AST in STZ-induced diabetic AD in light and dark chamber test response changes. Data of TSLC levels are expressed as mean standard deviation (± SD); ‘n’ is 20 zebrafish per group. ^a^ *p* < 0.05 value versus the normal group. ^b^ *p* < 0.05 value versus the AD control group. *Abbreviation:* AD, Alzheimer’s disease; AST, astaxanthin; CL-82198, MMP-13 inhibitor; DP, donepezil; and TSLC, time spent in light chamber.

A single injection of STZ (350 mg/kg, *i.p.*; and 2.5 µL, *i.c.*) has been shown to lead to the significant (*p* < 0.05) development of diabetic CVD-associated AD and impairment of neurocognitive behavior, increasing the time spent in the light chamber (TSLC) values in comparison to the normal control group. AST exposure (10 and 20 and 30 mg/L) has been shown to have a potential ameliorative effect against STZ-induced diabetic AD-associated neurobehaviour changes. The AST results mimic the reference drugs, i.e., donepezil (DP, 1 mg/L)- and CL-82198 (10 μM)-exposed groups. The results of AST in the amelioration of STZ-induced diabetic AD are illustrated in Figure 2.

### 2.2. Effect of AST on STZ-Induced Diabetic CVD-Associated AD in Color Recognition Test Response Changes

**Figure 3:**
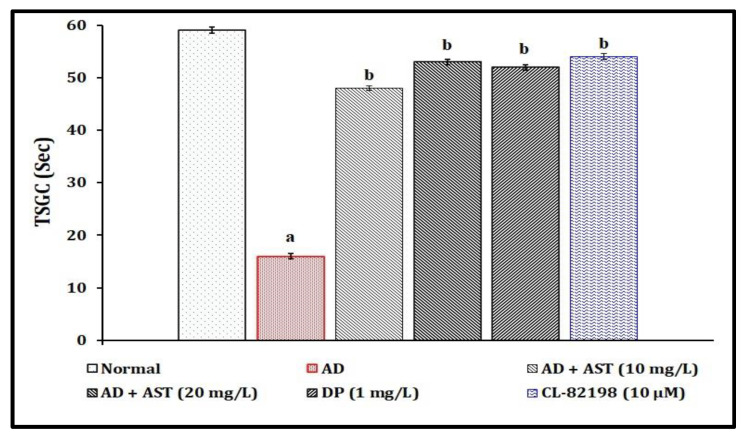
Role of AST in STZ-induced diabetic AD in color recognition test response changes. Data of TGSC levels are expressed as mean standard deviation (± SD); ‘n’ is 20 zebrafish per group. ^a^ *p* < 0.05 value versus the normal group. ^b^ *p* < 0.05 value versus the AD control group. *Abbreviation:* AD, Alzheimer’s disease; AST, astaxanthin; CL-82198, MMP-13 inhibitor; DP, donepezil; and TSGC, time spent in the green chamber.

The single injection of STZ (350 mg/kg, *i.p.*; and 2.5 µL, *i.c.*) has been shown to lead to the significant (*p* < 0.05) development of diabetic CVD-associated AD and impairment of neurocognitive behavior by reducing the TSGC values in comparison to the normal control group. The exposure of AST (10 and 20 and 30 mg/L) has been shown to have a potential ameliorative effect against STZ-induced diabetic AD-associated neurobehaviour changes. The AST results mimic the reference drugs, i.e., DP (1 mg/L)- and CL-82198 (10 μM)-exposed groups. The results of AST in the amelioration of STZ-induced diabetic AD are illustrated in Figure 3.

### 2.3. Effect of AST on STZ-Induced Diabetic CVD-Associated AD in T-Maze Test Response Changes

**Figure 4:**
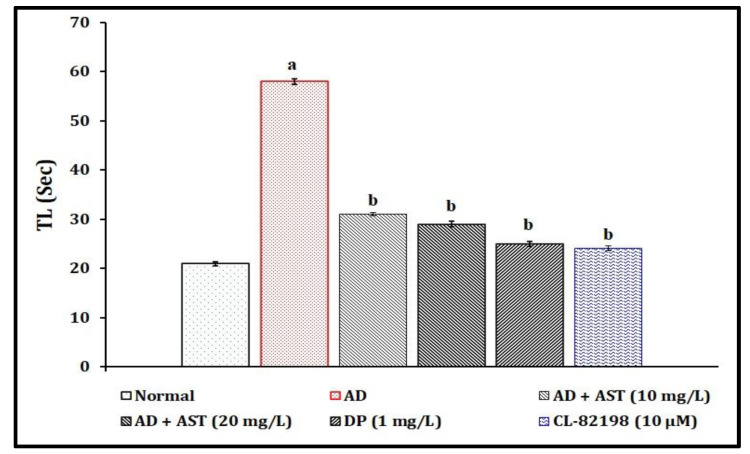
Role of AST in STZ-induced diabetic AD in T-maze test response changes. Data of TL levels are expressed as mean standard deviation (± SD); ‘n’ is 20 zebrafish per group. ^a^ *p* < 0.05 value versus the normal group. ^b^ *p* < 0.05 value versus the AD control group. *Abbreviation:* AD, Alzheimer’s disease; AST, astaxanthin; CL-82198, MMP-13 inhibitor; DP, donepezil; and TL, transfer latency.

A single injection of STZ (350 mg/kg, *i.p.*; and 2.5 µL, *i.c.*) has been shown to be significant (*p* < 0.05) in the development of diabetic CVD-associated AD and impairment of neurocognitive behavior by raising the TL values in comparison to the normal control group. The exposure of AST (10 and 20 and 30 mg/L) has been shown to have a potential ameliorative effect against STZ-induced diabetic AD-associated neurobehaviour changes. The AST results mimic the reference drugs, i.e., DP (1 mg/L)- and CL-82198 (10 μM)-exposed groups. The results of AST in the amelioration of STZ-induced diabetic AD are illustrated in Figure 4. 

### 2.4. Effect of AST on STZ-Induced Alterations in Fasting Blood Glucose Level

A single injection of STZ (350 mg/kg, *i.p.*; and 2.5 µL, *i.c.*) has been shown to have a significant effect on fasting blood glucose levels in comparison to the normal control group. The exposure of AST (10 and 20 and 30 mg/L) has been shown to have a potential ameliorative effect against STZ-induced fasting blood glucose level changes. The results of AST mimicked those of the reference drugs, i.e., DP (1 mg/L)- and CL-82198 (10 μM)-exposed groups. The results of AST regarding the amelioration of STZ-associated changes in fasting blood glucose levels are tabulated in Table 1.

### 2.5. Effect of AST on STZ-Induced Alterations in Evans Blue Dye (EBD) Concentration

**Figure 5:**
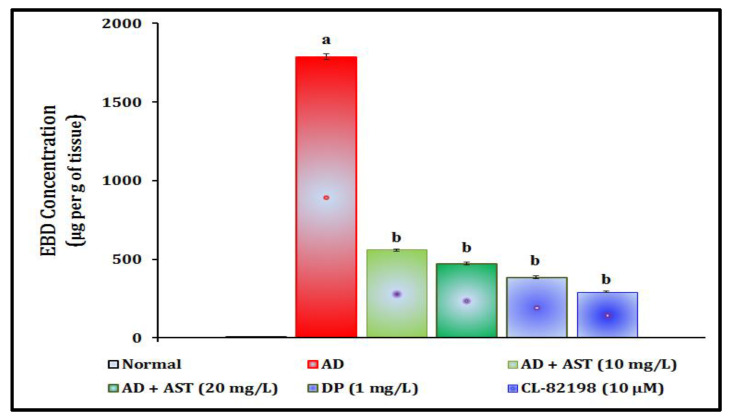
Role of AST in STZ-induced Evans blue dye concentration changes. Data of EBD concentration levels are expressed as mean standard deviation (± SD); ‘n’ is 20 zebrafish per group. ^a^ *p* < 0.05 value versus the normal group. ^b^ *p* < 0.05 value versus the AD control group. *Abbreviation:* AD, Alzheimer’s disease; AST, astaxanthin; CL-82198, MMP-13 inhibitor; DP, donepezil; and EBD, Evans blue dye.

The single administration of STZ (350 mg/kg, *i.p.*; and 2.5 µL, *i.c.*) has been shown to lead to a significant increase in the level of tissue extravasation as an indication of increases in the Evans blue dye concentration in comparison to the normal control group. AST exposure (10 and 20 and 30 mg/L) has been shown to potentially ameliorate the STZ-induced changes presented above. The AST results mimicked those of the reference drugs, i.e., DP (1 mg/L)- and CL-82198 (10 μM)-exposed groups. The results of AST regarding the amelioration of STZ-induced Evans blue dye concentration changes are illustrated in Figure 5.

### 2.6. Effect of AST on STZ-Induced Diabetic CVD-Associated AD-Mediated Tissue Marker Changes

The single administration of STZ (350 mg/kg, *i.p.*; and 2.5 µL, *i.c.*) has been shown to lead to a significant (*p* < 0.05) increase in tissue nitrite, neuronal amyloid beta-peptides, MMP-13, and AChE activities in comparison to the normal control group. AST exposure (10 and 20 and 30 mg/L) has been shown to potentially ameliorate the STZ-induced tissue biomarker changes. The AST results mimicked those of the reference drugs, i.e., DP (1 mg/L)- and CL-82198 (10 μM)-exposed groups. The results of AST regarding the amelioration of STZ-induced tissue biomarker changes are tabulated in Table 2.

## 3. Discussion

In the present study, a single injection of STZ (350 mg/kg, *i.p.*; and 2.5 µL, *i.c.*) led to the significant elevation of TSLC values in light and dark chamber tests, reduced the TSGC values in color recognition test, and raised the TL values in a T-maze test. Furthermore, STZ increased the fasting blood glucose levels, and tissue extravasation levels as indicated via the increase in Evans blue dye concentration level and tissue nitrite, as well as neuronal amyloid beta-peptide, MMP-13 and AChE activities. However, AST exposure (10 and 20, and 30 mg/L) and reference drugs, i.e., DP (1 mg/L) and CL-82198 (10 μM), were shown to ameliorate STZ-induced changes in the cognitive neurobehaviours and plasma and tissue biomarkers. Hence, AST plays a key role in the modulation of diabetic CVD and neurodegenerations.

AD is one of the major neurodegenerative disorders that alters cognitive functions. Experimentally, the overexpression of MMP-13 also causes AD, and the administration of MMP-13 inhibitors attenuates the cognitive dysfunction in AD transgenic mice via the beta-site of amyloid precursor protein cleaving enzyme 1 (BACE1) regulation [28]. Similar results were observed in the present study, where AST attenuated the diabetic CVD-associated AD (cognitive dysfunctions) via the inhibition of MMP-13 and AchE activities. AST also attenuated the CVD markers, i.e., extravasation (BBB damage and systemic leakage of Evans blue dye) and increased nitrite levels. These data indicate that AST regulates BBB integrity, similar to other experimental data [23]. Neuroinflammation in AD indicates the accumulation of beta-amyloid protein expression in experimental diabetic animals due to metabolic abnormalities in neuronal astrocyte function [29]. Similar results were shown in this study; AST reduces the aggregation of beta-amyloid protein and regulates AD pathology in experimental animals through reductions in cognitive dysfunction. AChE activity is a hallmark for the assessment of neuronal cholinergic neurotransmission and AchE activities, leading to a reduction in the acetylcholine contents in neuronal tissue and causing cognitive dysfunction in diabetic VaD and AD patients [30]. Carotenoids are also known to reduce neuronal acetylcholinesterase activity and enhance the acetylcholine contents in neuronal tissue [31,32].

AST has neuroprotective action against beta-amyloid-induced neuronal insulin resistance in AD models in rats [33]. Clinically, AST is reported to produce neuroprotective action [34]. However, the pathophysiological mechanism of AD has not been fully explained. Chronic hyperglycemic conditions are known to enhance the elevation of MMP-13 protein expression in neuronal tissue, which causes neurovascular damage [35,36]. The inhibition of MMP-13 by natural carotenoids attenuates diabetic neurovascular damage and cognitive dysfunction [35,37]. The natural marine xanthophyll type of carotenoid, i.e., AST, possesses potential neurovascular protective actions in vitro and in vivo against hyperglycemia via the inhibition and regulation of MMPs and MMP-associated signaling pathways [26,27,38,39,40,41]. AST enhanced neuroprotection and significantly attenuates neuroinflammation in an experimental animal model [42] due to its multi-targeted molecular pathways and regulatory actions, including MMP inhibition [41,43]. The present results also show that AST produces a potential ameliorative effect against diabetic CVD-associated AD via the inhibition of MMP-13 and regulation of other molecular pathways. Based on these research outcomes, AST can be used as a neurovascular modulating medicine for various cerebrovascular and metabolic disorders. Furthermore, AST can potentially regulate oxidative-stress-induced mitochondrial dysfunction in the nervous system [44,45]. Mitochondrial dysfunction is a key player in the progression of AD via alterations in matrix metalloprotease activity [46]. However, this study was carried out in lower vertebrate models, as evidenced by the biochemical estimations. This may be one of the major limitations of this study. Therefore, more extensive studies are required, exploring the complete therapeutic potential in higher vertebrate models with a combination of MMP-13 and AST molecules to reveal the actions of the molecular signaling pathways, i.e., mitochondrial targeted actions. This investigation is in progress.

## 4. Materials and Methods

### 4.1. Animals

Eight-month-old disease-free adult male zebrafish were used in the current research work. The zebrafish were housed in a 10-liter aquarium, which was filled with potable drinking water. The conditions of the zebrafish home tank were conditioned with aeration with an aerator, the water temperature was maintained at 25 ± 0.5 °C with a thermostatic device system, and light and dark cycles, i.e., 14:10 h, were maintained with an automatic timer controller-associated lighting system in the complete experimental protocol. The acclimatization period, i.e., 2 weeks, was applied to allow for the adaptation of zebrafish to the newer environmental setup. The experimental procedures were started after the completion of the acclimatization period. The neurobehavior observations, i.e., light and dark chamber test, color recognition test, and T-maze test were assessed between 09.00 a.m. and 01.00 p.m. (to avoid the influence of hormonal and floating food-associated changes in neurobehaviours) in all the animals involved in the diabetic CVD-associated AD protocol. Floating food was supplied after the completion of neurobehaviour assessments.

### 4.2. Drugs and Chemicals

AST, Congo red-Amyloid-beta, 5,5-dithibis(2-nitrobenzoic acid), Evans blue, and streptozotocin (STZ) were procured from Sigma Chemical, India. Donepezil was obtained from Cipla Limited, Mumbai, India. The MMP-13 inhibitor, i.e., CL-82198 was acquired from MedChemExpress, BioSynTech Malaysia Group Sdn Bhd, Selangor, Malaysia. The nitrite, amyloid beta-peptide, MMP-13 activity, AChE activity, and total protein ELISA assay kit was acquired from Thermo Scientific, Selangor, Malaysia. All the chemical reagents were used of analytical grade.

### 4.3. Induction of Diabetic CVD-Associated AD

Diabetes was produced by injecting streptozotocin (STZ, 350 mg/kg) intraperitoneally (*i.p.*), as described by Wang et al. [47] and Paramakrishnan et al. [35]. In a nutshell, zebrafish were sedated with an ice-cold solution at 5 °C. A total of 50 microliters of STZ stock solution (7 mg of STZ in 1 mL of normal saline) was injected using an insulin syringe. On the third day, blood drops were collected from the zebrafish by the gentle application of a lancet needle near the side midlines of the dorsal and anal fins of the zebrafish. The fasting blood glucose level in zebrafish was assessed using a glucometer, as described by Mohammadi et al. [48]. The blood glucose levels were raised above 15 mM glucose, which is considered to create diabetic conditions in zebrafish. Diabetic zebrafish were used for the induction of CVD by the intracerebral (*i.c.*) administration of STZ, a technique explained by Skaggs et al. [49]. In brief, 2.5 µL of STZ stock solution was injected via *i.c.* route using a dissecting stereomicroscope and Hamilton syringe (30-gauge) under anesthetic conditions. After injection, animals were allowed to recover in recovery tanks with environmental conditions similar to their home cage.

### 4.4. Experimental Protocol

A total of six groups of animals were used in the present study. Each group comprised 20 male adult zebrafish animals (*n* = 20).

➢ Group 1: This group functioned as the normal control group and were not administered with any drugs.
➢ Group 2: This group functioned as a diabetic CVD-induced AD control group. The induction of diabetic CVD-associated AD was described in the previous sections.
➢ Groups 3 and 4: This group was exposed to AST doses, i.e., 10 and 20 mg/L for 21 consecutive days (from day 03) in diabetic AD-induced animals.
➢ Groups 5: This group was exposed to donepezil (DP, 1 mg/L; for 21 consecutive days from day 03) in diabetic AD-induced animals.
➢ Groups 6: This group was exposed to CL-82198 (10 μM for 21 consecutive days from day 03) in diabetic AD-induced animals.

All the neurobehavioral parameters were assessed on the 21st day of drug administration. On the 22nd day, the blood samples were collected again, using the technique presented by Pedroso et al. [50], for blood glucose estimation. Thereafter, zebrafish were sacrificed, and their brain tissue was isolated for the quantification of tissue nitrite, amyloid beta-peptide aggregation, MMP-13 activity, AChE activity, and total protein.

### 4.5. Assessment of Neurobehavioural Tests

All the behavioral parameters, i.e., light and dark chamber test, color recognition test, and T-maze test, were carried out on the 21st day. Thereafter, fish were sacrificed, and plasma and tissue samples were collected for biomarker estimation.

#### 4.5.1. Assessment of Light and Dark Chamber Test

Special memory was assessed in zebrafish using the light and dark chamber test, as per the report of Dubey et al. [51], with the slight changes made by Rishitha and Muthuraman [52]. In brief, the dimensions of the apparatus were as follows: 10 cm in width, 20 cm in length, and 23 cm in height transparent glass tank. The water was filled up to 12 cm in height. The tank was partitioned (10 cm) into two equal vertical segments. The right half was covered with black paper to create a dark chamber compartment. The left half was received natural light with 50 Lux intensity. The cognitive function was assessed by assessing TSLC within one minute of exposure to each test chamber. If the animal does not travel to the light room, it is thought to have a weak memory, but moving to the light chamber indicates an improvement in memory function.

#### 4.5.2. Assessment of Color Recognition Test

The spatial memory function is also assessed by a color recognition test, as per the report of Dubey et al. [51], with the minor changes of Muthuraman et al. [53]. In brief, the color recognition test chamber was made of transparent glass with dimensions of 20 cm in length, 10 cm in width, and 10 cm in height. The chamber was divided into two equal vertical halves. The right side of the chamber was covered in red glass and served as a punishment cell. The left side of the chamber was covered in green glass and served as the reward chamber. The water was poured to a height of 5 cm. The cognitive function was assessed by evaluating TSGC within one minute of exposure. The animal was placed at the center of the chamber. If the animal did not migrate to the green room, it was assumed to have a weak memory, whereas if the animal preferred the green chamber, it was assumed that the animal’s memory function had improved.

#### 4.5.3. Assessment of T-Maze Test

Zebrafish memory functions were assessed in the T-maze test, as per the report of Buccafusco [54], with the slight modifications of Colwill et al. [55]. In brief, the T-maze apparatus comprised a transparent glass plate with two small arms of 10 cm in length, 6 cm in width, and 10 cm in height. Each arm was covered with different color plates. The right arm was covered with a red glass plate and the right arm was covered with green glass plates. The long middle arm’s dimensions were maintained, i.e., 20 cm in length, 10 cm in breadth, and 10 cm in height, and this was regarded as a home chamber. It was maintained as non-transparent yellow glass plates. The water was filled up to 3 cm in height. The adaptation of zebrafish to this T-maze environment was ensured by two minutes of exposure to this test device. If the animal reached a red chamber, the punishment stimuli were applied by stirring water in that chamber using a glass rod, whereas if the animal reached a green chamber, the reward stimuli were given by placing floating food. The next day, zebrafish were placed in the long arm’s corner as a beginning point and allowed to reach the objective point, which was the green chamber. The time it took to reach the green chamber was recorded as transfer latency (TL) within one minute of exposure. If the animal showed a lower TL value, this was indicated as an enhancement of memory function.

### 4.6. Estimation of Cerebral Extravasation Using Evans Blue Dye

The changes in cerebrovascular function were estimated as an indication of cerebral extravasation using the Evans blue dye method, as per the report of Yen et al. [56]. In brief, on the 22nd day, six zebrafish were selected in each group for the assessment of cerebral extravasation. The zebrafish were sedated with an ice-cold solution at 5 °C. A stock solution of 2.5 µL of 4% Evans blue dye was injected into the caudal vein. The 4% Evans blue dye stock solution was made by dissolving 400 milligrams of Evans blue dye in 10 mL of normal saline solution (0.9% *w/v* sodium chloride). A total of 120 min post-dye-injection, zebrafish were anesthetized, brain tissue was collected as described by Lopez-Ramirez et al. [57], and the assessment of extravasated dye concentration was carried out as described by Uyama et al. [58]. The brain samples were dried and weighted before being homogenized with 1:3 *v/v* of 50% tri-chloroacetic acid (TCA, dissolved in normal saline) and centrifuged for 10 min at 10,000 rpm. To remove the proteins, the supernatant was further diluted with 1:300 *v/v* 50% TCA. The aliquot was then mixed with 1:3 *v/v* of 95% ethanol solution for a spectrofluorimetric analysis of Evans blue dye contents at 620 nm excitation and 680 nm emission wavelengths. The levels of tissue Evans blue concentration are expressed as µg per gram of tissue.

### 4.7. Estimation of Tissue Biomarkers

The changes in diabetic CVD-associated AD-induced nitrite, neuronal amyloid beta-peptide aggregation, brain MMP-13 activity, AChE activities, and total protein contents were quantified as described in commercially available kits. The levels of tissue nitrite are presented as µM per mg of protein. The aggregation of soluble neural amyloid beta-peptide are presented as pg per mg of protein. MMP-13 activity in the brain was measured in nanograms per milligram of protein. AChE activity in the brain was quantified as µM of acetylthiocholine hydrolyzed per mg of protein per minute.

### 4.8. Statistical Analysis

The data are all presented as mean standard deviation (SD). Using the Statistical Package for the Social Sciences (SPSS) version 25 software, data from behavioral assessments, Evans blue dye, and tissue biomarkers were statistically analyzed using one-way analysis of variance (ANOVA), followed by multiple comparison tests with Tukey’s multiple range test. Statistical significance was defined as a probability value of *p* 0.05.

## 5. Conclusions

Exposure to AST, DP, and CL-82198 attenuates the STZ-induced diabetic CVD-associated AD and cognitive dysfunctions in zebrafish animal species. Hence, AST possesses the ability to oppose diabetic CVD-associated AD due to its potential blood glucose regulation, reductions in tissue extravasation, reversal of the neuronal nitrite, prevention of β-amyloid protein accumulation, and inhibition of MMP-13 and AChE activities. Hence, the natural marine drug, i.e., AST, may be used for the management of AD patients due to its multi-targeted actions, including MMP-13 inhibition.

## Figures and Tables

**Table 1:** Effect of AST on STZ-induced fasting blood glucose level changes.

Groups	Fasting Blood Glucose (mg/dL)
Normal	69.2 ± 1.1
AD	147.8 ± 2.4 ^a^
AD + AST (10 mg/L)	84.2 ± 1.9 ^b^
AD + AST (20 mg/L)	73.1 ± 1.5 ^b^
AD + DP (1 mg/L)	72.8 ± 1.4 ^b^
AD + CL-82198 (10 μM)	75.3 ± 1.7 ^b^

Data of fasting blood glucose levels are expressed as mean standard deviation (± SD); ‘n’ is 20 zebrafish per group. ^a^ *p* < 0.05 value versus the normal group. ^b^ *p* < 0.05 value versus the AD control group. *Abbreviation:* AD, Alzheimer’s disease; AST, astaxanthin; CL-82198, MMP-13 inhibitor; and DP, donepezil.

**Table 2:** Effect of AST on STZ-induced brain tissue biomarker changes.

Groups	Nitrite (µM/mg of protein)	β-Amyloid Peptide (pg/mg of protein)	MMP-13 (ng/mg of protein)	AChE (µM/mg of protein/Min)
Normal	58.2 ± 1.1	2.1 ± 1.3	11.4 ± 0.9	16.7 ± 1.6
AD	137.7 ± 1.3 ^a^	19.3 ± 0.9 ^a^	48.6 ± 0.6 ^a^	49.1 ± 1.2 ^a^
AD + AST (10 mg/L)	76.3 ± 1.2 ^b^	7.2 ± 0.7 ^b^	24.3 ± 0.3 ^a^	26.4 ± 1.3 ^b^
AD + AST (20 mg/L)	71.1 ± 0.9 ^b^	6.3 ± 0.6 ^b^	19.1 ± 0.6 ^b^	22.1 ± 0.9 ^b^
AD + DP (1 mg/L)	69.5 ± 1.1 ^b^	4.6 ± 1.1 ^b^	17.5 ± 0.7 ^b^	19.2 ± 1.2 ^b^
AD + CL-82198 (10 μM)	63.4 ± 0.8 ^b^	3.4 ± 1.2 ^b^	14.4 ± 0.4 ^b^	17.8 ± 1.4 ^b^

Data of EBD concentration levels are expressed as mean standard deviation (± SD); ‘n’ is 14 zebrafish per group. ^a^ *p* < 0.05 value versus the normal group. ^b^ *p* < 0.05 value versus the AD control group. *Abbreviation:* AChE, acetylcholinesterase; AD, Alzheimer disease; AST, astaxantin; β-amyloid peptide, soluble form of amyloid-beta peptide1-42 (Aβ_1-42_); CL-82198, MMP-13 inhibitor; DP, donepezil; and MMP-13, matrix metalloproteinase-13.

## Data Availability

Data are contained within the article.
